# *In vitro* propagation of XXY human Klinefelter spermatogonial stem cells: A step towards new fertility opportunities

**DOI:** 10.3389/fendo.2022.1002279

**Published:** 2022-09-28

**Authors:** Guillermo Galdon, Nicholas A. Deebel, Nima Pourhabibi Zarandi, Darren Teramoto, YanHe Lue, Christina Wang, Ronald Swerdloff, Mark J. Pettenati, William G. Kearns, Stuart Howards, Stanley Kogan, Anthony Atala, Hooman Sadri-Ardekani

**Affiliations:** ^1^ Wake Forest Institute for Regenerative Medicine (WFIRM), Winston-Salem, NC, United States; ^2^ Facultad de Medicina, Escuela de doctorado, Universidad de Barcelona, Barcelona, Spain; ^3^ Department of Urology, Wake Forest School of Medicine, Winston-Salem, NC, United States; ^4^ Division of Endocrinology, The Lundquist Institute at Harbor-University of California, Los Angeles (UCLA) Medical Center, Los Angeles, CA, United States; ^5^ Section of Medical Genetics, Department of Pathology, Wake Forest School of Medicine, Winston-Salem, NC, United States; ^6^ AdvaGenix and Johns Hopkins Medicine, Baltimore and Rockville, MD, United States

**Keywords:** spermatogonia, stem cell, germ cell transplantation, fertility preservation, Klinefelter Syndrome, male infertility, cell culture

## Abstract

Klinefelter Syndrome (KS) is characterized by a masculine phenotype, supernumerary sex chromosomes (47, XXY), and impaired fertility due to loss of spermatogonial stem cells (SSCs). Early testicular cryopreservation could be an option for future fertility treatments in these patients, including SSCs transplantation or *in vitro* spermatogenesis. It is critically essential to adapt current *in vitro* SSCs propagation systems as a fertility option for KS patients. KS human testicular samples (13,15- and 17-year-old non-mosaic KS boys) were donated by patients enrolled in an experimental testicular tissue banking program. Testicular cells were isolated from cryopreserved tissue and propagated in long-term culture for 110 days. Cell-specific gene expression confirmed the presence of all four main cell types found in testes: Spermatogonia, Sertoli, Leydig, and Peritubular cells. A population of ZBTB16^+^ undifferentiated spermatogonia was identified throughout the culture using digital PCR. Flow cytometric analysis also detected an HLA^-^/CD9^+^/CD49f^+^ population, indicating maintenance of a stem cell subpopulation among the spermatogonial cells. FISH staining for chromosomes X and Y showed most cells containing an XXY karyotype with a smaller number containing either XY or XX. Both XY and XX populations were able to be enriched by magnetic sorting for CD9 as a spermatogonia marker. Molecular karyotyping demonstrated genomic stability of the cultured cells, over time. Finally, single-cell RNAseq analysis confirmed transcription of ID4, TCN2, and NANOS 3 within a population of putative SSCs population. This is the first study showing successful isolation and long-term *in vitro* propagation of human KS testicular cells. These findings could inform the development of therapeutic fertility options for KS patients, either through *in vitro* spermatogenesis or transplantation of SSC, *in vivo*.

## Introduction

Klinefelter Syndrome (KS) is a classically underdiagnosed cause of male infertility characterized by a male phenotype and altered karyotype, usually presenting as non-mosaic 47 XXY (1). However, variants have been described with additional X chromosomes and different degrees of mosaicism. Recent studies using non-invasive genetic diagnostic technology estimated KS may affect one of every 600-1000 males born (2, 3). Hence, KS is now considered the most common chromosomal cause of male infertility.

The pathophysiological mechanisms underlying male infertility in KS patients are not yet entirely understood. However, several studies have attempted to describe KS testicular morphology from the fetus into adulthood (4–6). Testicular morphology remains largely unaffected in prepubertal patients. During puberty, when testicular fibrosis accelerates, distortion of the tubular architecture was seen (7). By the end of puberty, over 95% of KS patients are azoospermic (8). Leydig cell hyperplasia occurs in the interstitial space in response to elevated LH. Despite Leydig cell hyperplasia, KS patients commonly present with low or low normal range serum testosterone levels. Nevertheless, the degree of testicular fibrosis differs from patient to patient. Foci of active spermatogenesis have been found in KS patients with testicular fibrosis. Current literature shows that only 8% of KS patients have sperm present in the ejaculate (9). These KS patients with adequate sperm in the ejaculate can achieve natural conception with euploid offspring suggesting that cells that complete meiosis may develop into normal gametes (10). The current gold standard of care for treatment of infertility in KS patients is micro-TESE (microscopic testicular sperm extraction) with a reported 44% success rate in testicular sperm retrieval (11, 12), 43% pregnancy rate, and 43% birth rate from *in vitro* fertilization (IVF) or intracytoplasmatic sperm injection (ICSI). No other fertility options for KS patients are currently available.

Since the development of prenatal genetic diagnostic techniques (e.g. Amniocentesis, Chorion Villus Sample, Cell-Free-Fetal DNA determination, Quantitative Fluorescence PCR, Multiplex Ligation Dependent Probe Amplification, Preimplantation Genetic Screening), it is now possible to diagnose and follow KS patients in the prepubertal period, providing social support and enhanced education for preservation of occult sperm that would provide fertility options, should infertility develop. Several reports showed that in patients from whom no spermatozoa could be retrieved by microTESE, viable spermatogonia, including SSCs, may be found in testicular biopsies (13, 14). SSCs are a subpopulation of undifferentiated spermatogonia present in the testis. Their primary function is both continuous self-renewal and differentiation into germ cells committed to undergo spermatogenesis (15). It has been hypothesized that SSCs should be capable of complete restoration of spermatogenesis following transplantation into infertile patients (16). This hypothesis has been supported by several different animal studies (17–22). However, successful SSCs transplantation in humans has yet to be accomplished (23). Experimental SSCs-based therapies may potentially treat KS patients’ infertility, even in those patients with unsuccessful TESE. Testicular tissue banking has been implemented in KS patients to provide a source of spermatogonia for new fertility treatments (24, 25). As previously mentioned, germ cell loss in KS accelerates with the onset of puberty. Therefore, retrieval of viable spermatogonia in testicular biopsy is higher in peripubertal than in adult KS patients (8). About 25% of KS patients are diagnosed before puberty (1), and storing testicular tissue from these patients is not a standard practice. Our Wake Forest Baptist School of Medicine group has established an experimental testicular tissue banking for boys and men with a high risk of infertility. KS is one of the approved indications (26). Participating patients are given the option to donate a portion of their testicular samples for research voluntarily.

Our setting provides a unique opportunity to explore the ability to isolate and propagate KS testicular cells from testicular biopsy performed before puberty. Expanding the number of KS testicular cells *in vitro* would be critically important when considering autologous cell-based fertility treatments. It would also provide material for research to preserve and enhance fertility in KS patients, and improve our understanding of why XXY males lose germ cells at puberty. Our previous work tested SSC isolation and culture on 41 XXY KS mice (27–30). Cells were successfully propagated in culture for up to 120 days while expanding 650,000-fold in number. Moreover, characteristic phenotypes of all four common cell types were maintained in culture, including a population of putative SSCs (30). Similar findings are now confirmed using human frozen testicular tissue from KS patients.

## Material and methods

### Patients

Selected Klinefelter adolescent patients eligible for micro-Testicular Sperm Extraction (mTESE) were offered the opportunity to store their testicular tissue for potential stem cell therapy at Wake Forest Baptist experimental testicular tissue bank under IRB approved protocols at Wake Forest School of Medicine (IRB00021686 and IRB00061265). Surgery was performed by experienced pediatric and adult reproductive urologists (26). Intraoperative testicular tissue examination to evaluate the presence of sperm was performed by the clinical embryologist. The protocol included several rinses, microbiology testing, and histology tissue processing. Testicular samples were dissected into 2-4mm^3^ portions and cryopreserved in 1ml cryovials using cryoprotectant solution: Hank Balances Saline Solution 5% Human Serum Albumin (CSL Behring LLC) 5% DMSO (Mylan Institutional). A controlled rate freezing machine was used to freeze the samples following the in house validated protocol (26, 31). Cryovials were transferred into vapor nitrogen tanks at the Manufacturing and Development Center (MDC) of Wake Forest Institute for Regenerative Medicine (WFIRM) for long-term storage. A maximum of 20% of stored tissue was used in this study. The remaining portion (80%) was kept for future clinical use.

A portion of the testicular biopsies was fixed in 4% Formalin, 4% Paraformaldehyde, and Bouin fixatives. Tissue was then processed, paraffinized, and mounted by a clinical pathology lab for staining and histology.

Hematoxylin-Eosin staining and immunohistochemical staining with PGP 9.5 (UCHL1) as a spermatogonia marker were performed on 5 micron thick tissue sections using an automated stainer. Immunostaining was performed using the Leica Microsystems Bond 3 autostainer at the Wake Forest University clinical pathology laboratory. After 20 minutes of antigen retrieval using Bond Epitope Retrieval Solution 2 (ER2), the primary antibody (PA0286; mouse monoclonal PGP9.5 (UCHL1)) was incubated for 15 minutes at room temperature. To detect and visualize the antigen, a Bond Polymer Refine Detection kit (peroxide block, post-primary, polymer reagent, DAB chromogen/Leica, DS9800, followed by hematoxylin counterstain) was used. Counterstaining of hematoxylin identified the nucleus of cells. Isotype control (mouse IgG) was used as a negative control for the primary antibody. Age-matched controls (testes biopsies from 46 XY patients *via* National Disease Research Interchange, NDRI) were used to compare testicular morphology. Microscopic images were acquired using LEICA DM4000B microscope, Olympus camera DP73, and Olympus Cellsens software.

### Cell isolation, culture, and cryopreservation

Testicular cells were isolated from cryopreserved testicular tissue. The process was performed under pre-clinical Good Laboratory Practice (GLP)-conditions following a protocol previously described by our group (30, 32, 33) with some modifications for potential clinical application of human SSCs isolation and culture. Selected cryovials were thawed uniformly by immersion in warm water. Immediately after, DMSO was washed out from the tissue using 1x MEM 8µg/ml DNAse (Roche). External connective tissue was removed, and single seminiferous tubules were dissected apart using a dissecting microscope (Leica S6D) and jeweler tweezers.

Tissue was transferred into a 1.5 ml Eppendorf tube due to the small sample size and resuspended in an enzymatic digestion solution made of: 1x MEM; 12µg/ml DNAse (Roche); 0.4 PZU/ml Collagenase NB4 Standard Grade (SERVA); 0.02 DMCU/ml Natural Protease NB (SERVA). Samples were incubated in a shaking water bath at 120 rpm and 32°C. The enzyme mix was exchanged after every hour of incubation. Tubules and cells were pelleted by centrifugation at 16g for 5 min. A sample of the pellet was examined under the microscope, and the process was repeated with fresh enzymatic solution until disassociation of the tubules was confirmed by these examinations.

Once the enzymatic digestion disrupted the seminiferous tubules and many single floating cells were visible floating, the sample was pipetted up and down energetically to help release the remaining cells. The tube was then centrifuged for 5 min at 350g (1500 rpm) with a brake. The resultant supernatant was discarded, and pellets containing released cells were used for culture.

During the isolation process released tubular cells might form tight clumps. In these cases, it was helpful to additionally incubate the sample in 0.25% Trypsin-EDTA (FisherScientific-Gibco) at 37°C for 5-30 minutes, checking every 5 minutes until a single cell suspension was observed again. Then the sample was centrifuged again for 5 min at 350g (1500 rpm) with brake, trypsin supernatant was removed, and the pellet containing released cells was used for culture.

The final pellet of cells was resuspended on MEM 10% FBS for culture. This culture media was used to improve the initial attachment of cells. Hematocytometer should be used to assess cell numbers in the final sample and all the previously removed supernatants to ensure no cell is wasted. Viable cells were seeded at 10.000 cells/cm^2^ on plastic plates (Falcon) and kept in an incubator constant 37°C 5% CO2 in a room with positive air pressure GLP conditions. The next day, culture media was switched from MEM 10% FBS into an enriched StemPro medium, **Table 1** (32, 33), previously reported to support testicular cell propagation *in vitro*.

**Table 1:** Stempro Complete culture media composition.

Reagent	Company	Catalog #	Final concentration
**Stem Pro-34 SFM**	Invitrogren	10639-011	–
**Stem Pro Supplement**	Invitrogren	10639-011	–
**Bovine Albumine**	Roche	1.07E+10	5 mg/ml
**D(+) Glucose**	Sigma	G7021	6 mg/ml
**Ascorbic acid**	Sigma	A4544	1x 10-4
**Transferrin**	Sigma	T1147	100 μg/
**Pyruvic acid**	Sigma	P2256	30 mg/ml
**d-Biotin**	Sigma	B4501	10 μg/ml
**2-beta Mercatoethanol**	Sigma	M7522	5x 10-5
**DL-lactic acid**	Sigma	L4263	1 μl/ml
**MEM-non essential**	Invitrogen	11140-035	10 μl/ml
**Stem Pro Supplement**	Invitrogen	10639-011	26 μl/ml
**Insulin**	Sigma	I1882	25 μg/ml
**Sodium Selenite**	Sigma	S1382	30 nM
**Putrescine**	Sigma	P7505	60 μM
**L-Glutamine**	Invitrogen	25030-024	2 mM
**MEM Vitamine**	Invitrogen	11120-037	10 μl/ml
**b-Estradiol**	Sigma	E2758	30 ng/ml
**Progesterone**	Sigma	P8783	60 ng/ml
**Human EGF**	Sigma	E9644	20 ng/ml
**Human bFGF**	Sigma	F0291	10 ng/ml
**Human LIF**	Chemicon	LIF1010	10 ng/ml
**GDNF**	Sigma	G1777	10 ng/ml
**FCS**	Invitrogen	10106-169	1%
**Pen/Strep**	Invitrogen	15140122	0,5%

During the culture, samples were checked every other day. The media was refreshed every 2-3 days. Cells were passaged and split when cell confluency approached 80% using 0.25% Trypsin-EDTA (FisherScientific-Gibco).

After every passage, when possible, a portion of cells were cryopreserved for backup in a 2ml cryotube (Sigma Aldrich). Cryopreservation media used was MEM 20% FBS and 8% DMSO, and Mr.Frosty (Nalgene) at -80˚C was used for slow freezing overnight. The next day, cryotubes were transferred into liquid nitrogen tanks for long-term storage.

### Quantitative reverse transcriptase polymerase chain reaction

RNA was extracted using RNEasy Minikit (Qiagen) from Snap Frozen tissue or cells. The quality and quantity of extracted RNAs were tested with a spectrophotometer (Nanodrop 2000, ThermoFisher). RNA was then converted to cDNA using Reverse Transcriptase Kit (Life Technologies) and through the following thermocycler (Simpli amp thermal cycler, life technologies) conditions: 25°C for 10min, 37°C for 120min, 85°C for 5 min, and 4°C hold.

The cDNA samples underwent PCR amplification using Taqman primers (**Table 2**) and applied Biosystems 7300 Real Time PCR system. The cycling conditions followed were 95˚C for 10 minutes, then 40 cycles of 95˚C for 15 seconds, and 60˚C for 1 minute.

**Table 2:** Taqman assay primers used for PCR.

Gene	Catalog #	Primer length
**UCHL1**	Hs00985157 m1	80
**ZBTB16**	Hs00957433 m1	65
**THY1 (CD90)**	Hs00174816 m1	60
**PRM1**	Hs00358158 g1	99
**GATA4**	Hs00171403 m1	68
**Clusterin**	Hs00971656 m1	93
**CD34**	Hs02576480_m1	63
**STAR**	Hs00264912 m1	85
**TSPO**	Hs00559362 m1	57
**CYP11A1**	Hs00897320 m1	81
**POLR2A**	Hs00172187 m1	61

All primers were chosen intron spanning and previously tested for not amplifying genomic DNA. A minus RT (water) control has been used to rule out any contamination in PCR mixtures (not shown in the gel picture, **Figure 4**). POLR2A was selected as a housekeeping gene, and the expression of genes were normalized to this gene; relative expression was determined with the Delta CT method.

Amplified cDNA from RT qPCR was later used for an Electrophoresis study on a gel to visualize the specific product bands. A 2% Agarose gel was used, given that our target primers were between 50-120 bp in length (**Table 2**). Ethidium Bromide was included in the gel formulation at a concentration of 5ul/100ml. After the Samples and DNA ladder were loaded, a 140V Voltage (Enduro power supply, Labnet) was applied for 20 mins when the DNA dye reached 2/3 of the total gel size. Images of the gel were taken using a UV light Camera system (Gel logic 200 imaging system).

### Digital reverse transcriptase polymerase chain reaction

Digital PCR is a molecular biology tool that evenly distributes sample cDNA along with RT PCR mix into independent microchip wells. Then independent thermocycling reactions are conducted separately in each well prior to fluorescent signal reading. Digital PCR is especially useful for small population assessment or rare events analysis. This system has been validated by both manufacturer and independent researchers (34), even in a clinical setting (35, 36).

To estimate the percentage of undifferentiated spermatogonia during the culture, we used a Digital PCR system (Quant Studio, Life Technologies) and Taqman assays labeled with VIC (for POLR2A as housekeeping gene) and FAM for target gene (ZBTB16). As each mammalian cell has a range of 10–30 pg of total RNA, we used the cDNA made from 50 ng of total RNA in 20 000 wells of a digital PCR chip for each assay.

The chips were loaded following the manufacturer’s protocol with commercially available dPCR Master Mix (Life Technologies), water, cDNA, and Taqman primers. cDNA for this project was obtained using retrotranscriptase reaction on RNA extracted from Snap Frozen cells. Cycling conditions were: 95˚C for 10 minutes, then 40 cycles of 95˚C for 15 seconds and 60˚C for 1 minute.

### Flow cytometry analysis and MACS-sorting

The population of putative spermatogonial stem cells in culture was estimated as HLA-ABC-/CD9+/CD49f+ population (37–41) using BD Accuri C6 Flow cytometry system without sorting. BD antibodies were used (**Table 3**) at a concentration of 5ul of antibody per 50.000 cells in 100ul Flow Cytometry buffer (PBS 1% FBS). Cells were incubated with the antibody for 30 minutes at room temperature and then washed with flow cytometry buffer. Obtained data were analyzed using BD Accuri software. Unstained cells and cells incubated with isotypes control antibodies (**Table 3**) were used to optimize channel compensation and negative control. In every condition, 10,000 events were evaluated.

**Table 3:** Antibodies used for Flow cytometry and Magnetic-activated cell sorting (MACS).

Antigen	Reactive species		Raised in	Flourochrome	Company	Catalog #
HLA-ABC	Anti-Human		Mouse	FITC	BD Biosciences	555552
CD9	Anti-Human		Mouse	PE	BD Biosciences	341637
CD49f (Integrin alpha 6)	Anti-Human		Mouse	APC	Thermofisher	17-0495-80
Mouse IgG				FITC	BD Biosciences	340755
Mouse IgG				PE	BD Biosciences	340756
Mouse IgG				APC	BD Biosciences	340754
FcR Blocking Reagent	Anti-Human			MACS Microbeads	Miltenyl Biotec	120-000-442
Anti-PE	Anti-Human			MACS Microbeads	Miltenyl Biotec	120-000-294

The same staining method was used in separate experiments to perform Fluorescence Activated Cell Sorting using BD FACS ARIA. Cells expressing HLA-ABC-/CD9+/CD49f+ were sorted and used for FISH analysis.

### Single-cell RNA sequencing

Cultured and cryopreserved testicular cells from XXY and XY adolescent individuals were sent from WFIRM to UCLA. Upon arriving at the Lundquist Institute of Harbor-UCLA Medical Center, testicular cells were revived and cultured in a 25 cm^2^ flask with 4 ml of enriched StemPro-34 medium/flask for five days. Cells were harvested, washed, resuspended in 0.04% BSA in PBS, and delivered to UCLA Technology Center for Genomics and Bioinformatics (TCGB). The cell concentration and viability were determined at TCGB using a countess II automated cell count. Per manufacturer recommendations, a single cell suspension (1000 cells/µL) from each sample was loaded onto the 10x Chromium chip and controller. Ten thousand cells were targeted for capture per sample. Cell capture and 10X single cell 3’ gene expression sequence library preparation were performed. The resultant library was sequenced using a NovaSeq 6000 SP (100 Cycles). Mapping, cell identification, and clustering analysis were performed using 10x Cell Ranger software at the UCLA-TCGB.

For the data analysis, raw count matrices generated from 10X Genomics and Cell Ranger were imported to the Seurat package in R, where cells were screened based on QC metrics (42). An object of class Seurat 19618 features across 9580 cells of XY testicular sample and 19284 features across 11845 cells of XXY sample were subject to data analysis. Data normalization, scaling, and feature selection was performed as described in the SeuratV3 procedure. These were followed by unsupervised cell clustering and a UMAP analysis (43, 44). Differential gene expression among clusters was determined in Seurat using a Wilcoxon rank sum test, and gene expression probability across clusters was visualized with VlnPot and FeaturePlot.

### X and Y chromosome fluorescent *in situ* hybridization

After cells in culture had been trypsinized, fifty thousand cells were cytospin for 10 min at 1000 RPM on glass poly-l-lysine slides (Cytopro, ELI Tech Biomedical Systems) using the cytopro 7620 cytocentrifuge system (Wescor). The slides were left to dry at room temperature overnight. In parallel, testicular tissue samples from the same patients archived in pathology were processed by for the same X and Y hybridization.

Slides were soaked in 2X saline sodium citrate (made from stock 20xSSC from ABBOTT/VYSIS Company) at 37°C for 35 minutes. Subsequently, cells were incubated in Pepsin (AVANTOR PERFORMANCE MATERIALS, INC 2629, company) 0.5mg/ml solution of HCl 0.1M at 37 degrees C for 35 minutes. Slides were then washed at room temperature in 1XPBS for 5 minutes, and Post-Fixation Solution (0.9% formaldehyde W/V; 4.5 mg/ml MgCl2 in PBS) was added for 5 minutes incubation at room temperature. Slides were re-washed at room temperature 1XPBS for 5 minutes, and the dehydration process was performed by submerging slides in increasing concentrations of ethanol (70%, 80%, 100%) for 1 minute each at room temperature.

A working solution of X and Y chromosome probes (EMPIRE GENOMICS Company kit 1:1:4 probe buffer dilution) was added to the sample, and slides were kept at 75°C for 5 minutes, followed by overnight incubation at 40°C (16 Hours minimum). The following day, slides were washed in 0.3% IGEPAL/solution (SCI-GENE) on 0.4X saline sodium citrate/for 2 minutes at 73°C and 0.1% IGEPAL (SCI-GENE) solution on 2X saline sodium citrate for 1 minute at room temperature.

Slides were finally mounted with ABBOTT/VYSIS DAPI II and coverslipped.

Imaging of the slides was performed using a Zeiss Axiophot microscope and Applied Spectral Imaging Software.

### Molecular karyotyping with next generation sequencing

The cryopreserved cultured cells were transported from WFIRM to AdvaGenix and Johns Hopkins Medicine lab. After DNA extraction, fifty to 100 ng of amplified DNA underwent library preparation (Thermo Fisher Scientific, Waltham, MA). First, the pooled DNA samples underwent enzymatic shearing to produce fragment sizes of approximately 200 bp. The DNA fragments were purified using an AMPure Bead (Beckman Coulter, Sharon Il) wash, stabilized by the ligation of adapters and barcodes at either end of each fragment, and size selected using a second AMPure Bead wash. Each DNA fragment was bound to one ion sphere particle (ISP) and amplified thousands of times using an emulsion PCR reaction. The positive template ISPs were recovered using Dynabeads MyOne Streptavidin CI bead washes (Invitrogen, Carlsbad, CA). Following recovery, sequencing primers and Ion Hi-Q sequencing polymerase were added to the samples, and the samples were loaded onto a sequencing chip and analyzed at a depth of 1X across the entire genome by a Personal Genome Machine (PGM) or S5 (Thermo Fisher Scientific, Waltham, MA). Sequencing data were processed by a Torrent Browser Server (Thermo Fisher Scientific, Waltham, MA) to provide initial sequencing information and ensure adherence to our required quality assurance metrics. The data was then transferred to an Ion Reporter Server (Thermo Fisher Scientific, Waltham, MA) for comprehensive data analysis and interpretation. The PGM or S5 sequencing provided a minimum of over 3.5 million reads with a median sequencing fragment length of 181 bp.

## Results

### Presence of undifferentiated spermatogonia in TESE negative KS patients’ testes

**Figure 1:**
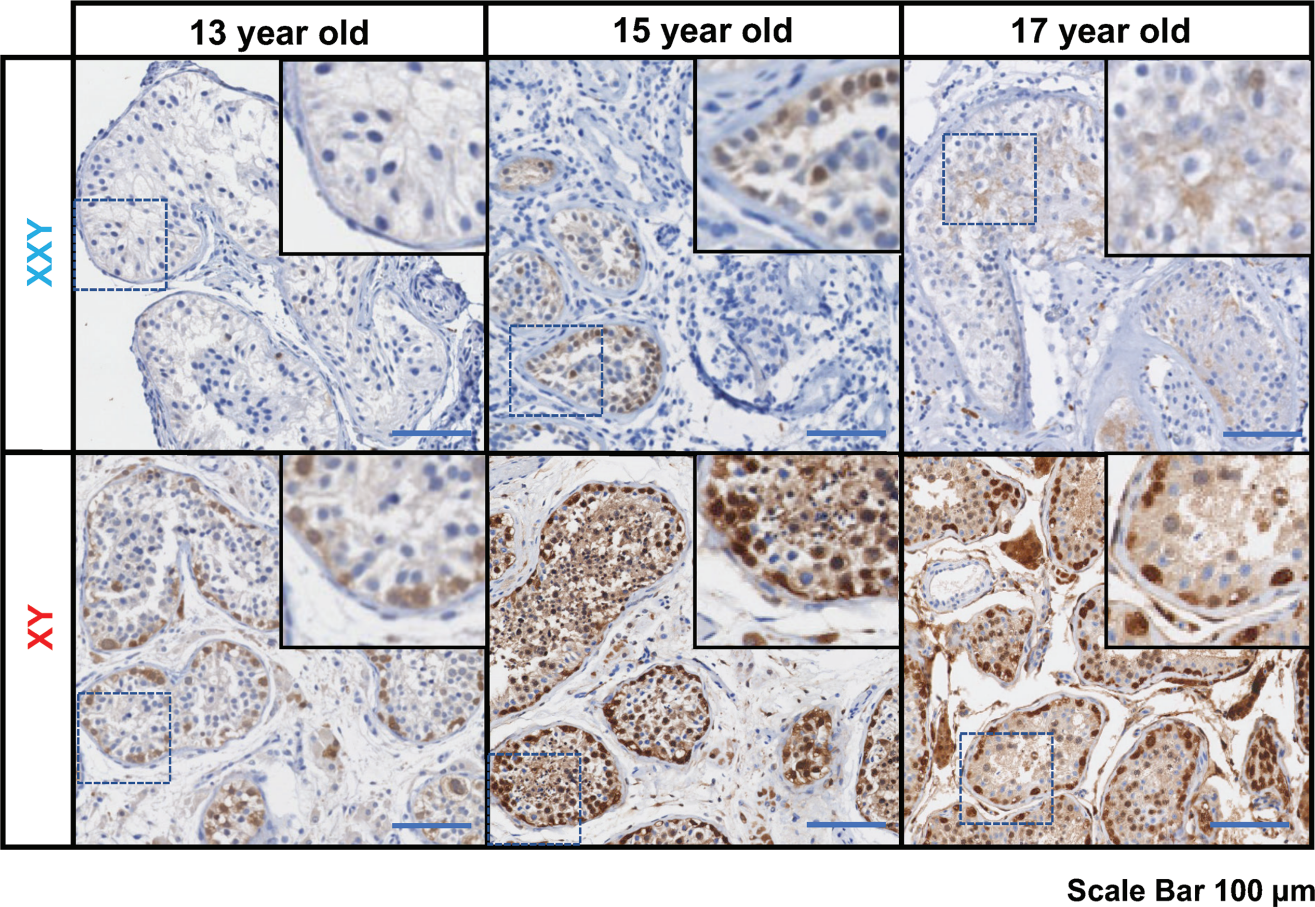
Immunostaining for undifferentiated spermatogonia marker UCHL1 (PGP 9.5), subjects’ ages: 13, 15 and 17 years old. On the top panel, three Klinefelter patients included in the study. On the bottom panel, three aged-matched controls. Scale bar 100µm.

Standard of care at Wake Forest testicular tissue bank includes histologic analysis and pathology review of all samples. Every KS patient case is discussed and compared with age-matched controls from our archives. Three KS patients enrolled in the testicular tissue bank were selected for this study (13 years old, 15 years old, and 17 years old). Pathology slides from testicular biopsy stained immunohistochemically for PGP 9.5 (UCHL1) and Hematoxylin-Eosin and compared with age-matched controls (**Figure 1**).

In all KS subjects, most seminiferous tubules were either hyalinized or only contained Sertoli cells. Rarely, some tubules contained the cells expressing the PGP 9.5, a marker for undifferentiated spermatogonia, with no further spermatogenic cells such as spermatocytes and spermatid. Leydig cell hyperplasia was prominent between tubules. No evidence of germ cell neoplasia or carcinoma *in situ* was observed. In contrast, age-matched controls presented a well-organized tubular architecture containing spermatogonia at different stages of differentiation into elongated spermatids.

Given that sperm were not found using micro TESE in any of the three patients (TESE negative), they were considered ideal candidates to benefit from SSCs isolation and culture strategy.

### Long term *in vitro* propagation of KS testicular cells

**Figure 2:**
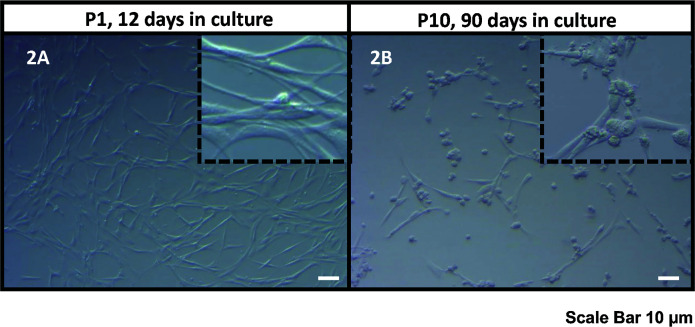
Bright field microscope images of the human KS testicular cells in culture at passage 1, 12 days in culture **(A)** and passage 10, 90 days in culture **(B)**. As it was also reported previously (30, 32, 33), the current culture system includes both spermatogonia and testicular somatic cells. Somatic cells usually attach earlier forming an extensive network of plain elongated cells. Then round spermatogonia cells attach forming clusters on top of somatic cell feeder layer (showed in inserts). Scale Bar 10 µm.

Compared to previous experiences by our group in isolating cells from human testes, KS samples presented as a different consistency. Tubules were thickly packed and more difficult to dissect. In our hands, pieces took between 2-2.5 h of enzymatic digestion to release cells, but it varied from sample to sample. Once the isolation method was tuned and optimized for KS tissue, cell isolation yield was comparable to XY age matched controls. Similarly, the morphology of cells in short- and long-term cultures was similar to previous testicular cells cultured from euploidy patients (**Figure 2**).

**Figure 3:**
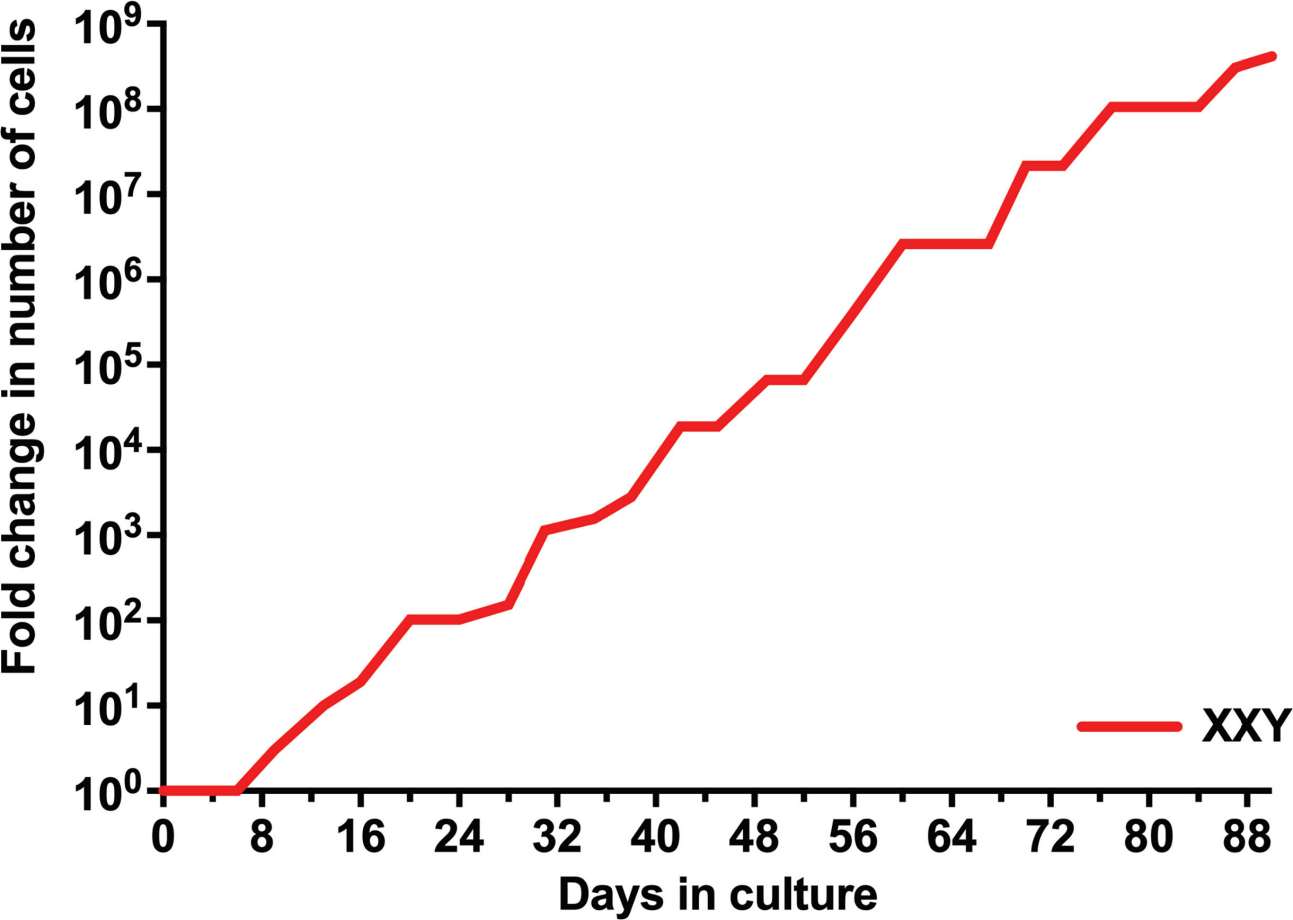
Evolution of the number of isolated human KS testicular cells in culture. Results show the average from KS subjects’ testicular tissue. The initial number of cells was standardized to 1 to better assess growth along time in the graph and compare subjects with different initial cell numbers.

Isolated testicular cells from KS patients remained viable in culture for more than 90 days. For the first eight days in culture, the number of cells did not significantly increase. Cells then started growing exponentially until the end of the study. The number of cells expanded more than 100 million fold within 90 days (**Figure 3**). Cell viability was always ≥ 95%, as determined by trypan blue staining at each passage. These findings are comparable to previous data from our group in propagating testicular cells from peripubertal euploid patients (32).

### Presence of undifferentiated spermatogonia and somatic cells in culture

After testicular cell isolation, a heterogeneous mixture of cells is expected in this culture system. Somatic cells are expected to quickly attach to the culture surface and form a feeding layer that provides an excellent environment for spermatogonia propagation. It is critically important to confirm the presence of all significant testicular cell types during the culture period, as a misbalance could prevent the other cell types from expanding.

**Figure 4:**
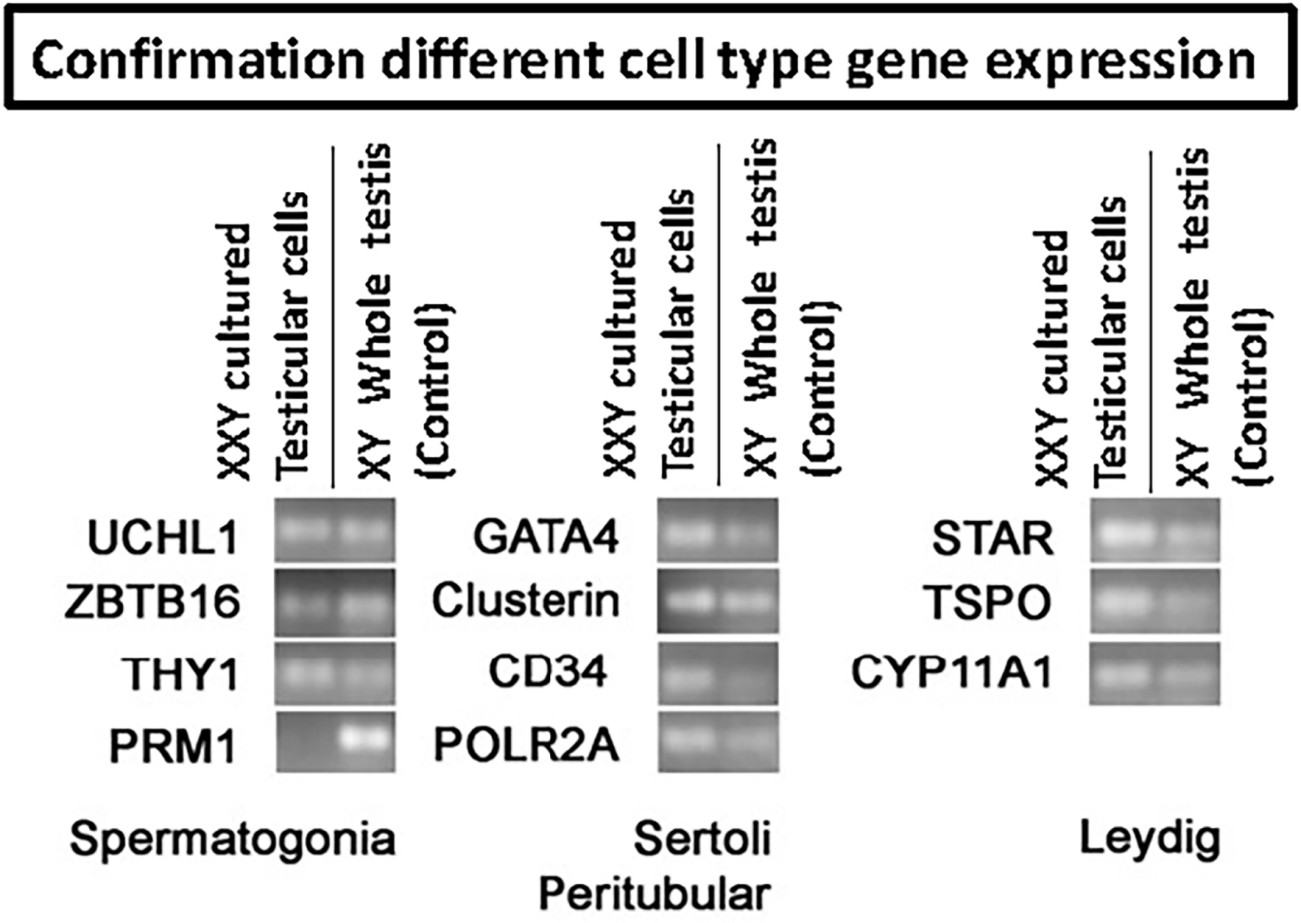
RT-PCR to confirm the presence of the four main testicular cells in culture based on cell-specific gene expression: spermatogonia (UCHL1, ZBTB16, THY1, PRM1), Sertoli (Gata4, Clusterin), peritubular (CD34) and Leydig (STAR, TSPO, CYP11A1). POLR2A was used as a housekeeping gene for internal control. Results confirmed the presence of all four common cell types. However, differentiated germ cell marker PRM1 remained negative, indicating the undifferentiated status of the germ cells in culture.

qPCR analysis was used to confirm the presence of characteristic gene expression from the main four testicular cell types expected in culture: undifferentiated spermatogonia (UCHL1, ZBTB16, and THY1), Leydig (TSPO, STAR, CYP11A1), Sertoli (GATA4, Clusterin) and peritubular cells (CD34) (**Figure 4**). A positive signal for all of these was obtained from cultured cells. Conversely, the expression of differentiated germ cell marker PRM1 remained negative (**Figure 4**). This demonstrated that spermatogonia remained undifferentiated and capable of maintaining a putative SSCs population suitable for transplantation.

**Figure 5:**
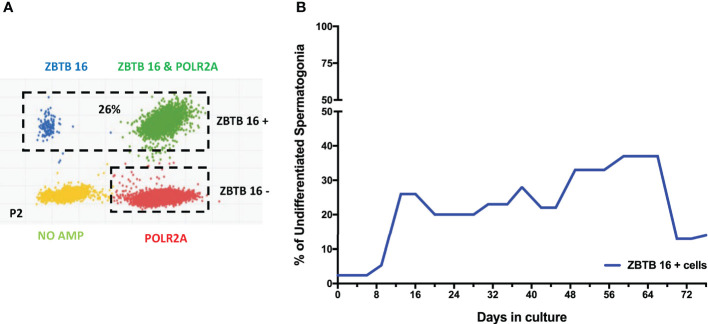
Digital PCR analysis was used to assess the population of undifferentiated spermatogonia expressing ZBTB16 (PLZF) marker and housekeeping gene POLR2A in testicular cells in culture: After 2nd passage from 15-year-old KS patient **(A)**. The same analysis was repeated at different time points. The graph shows the average undifferentiated spermatogonia population during the culture of testicular cells from KS patients **(B)**.

After confirming the phenotypes of cells present in the culture, the focus moved to determining the percentage of spermatogonia throughout the culture period. Digital PCR analysis was performed (**Figure 5**) on isolated testicular cells from frozen tissue with 2.3% of cells shown to be ZTBTB16^+^. This population grew during the initial stages of culture, climbing from 20- 37% between days 13-66. In the culture’s late stages, the percentage of undifferentiated spermatogonia dropped to 14% but remained present throughout the entire culture period (**Figure 5**). Therefore, our culture system for Klinefelter testicular cells propagated every cell type expected while maintaining a significant population of undifferentiated spermatogonia throughout the 90 days culture.

### The constant presence of SSCs during the culture

At the time of transplantation, only SSCs are expected to migrate to the basal membrane of the seminiferous tubules and restore spermatogenesis in azoospermic patients. So far, the scientific community has struggled to identify a single marker that could fully characterize this population of cells. Meanwhile, a combination of HLA-ABC -/CD9 +/CD49f + has been postulated as markers for the enrichment of SSCs and predicting therapeutic success following transplantation (38–41, 45).To assess putative SSCs in culture, flow cytometry analysis was utilized.

**Figure 6:**
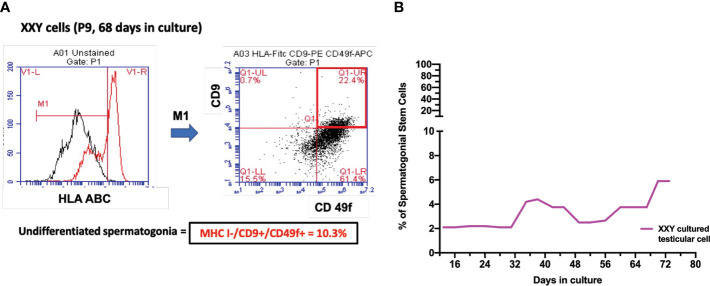
**(A)** Spermatogonial stem cells (SSC) population was estimated by combining HLA-/CD9+/CD49f+ markers on Flow Cytometry analysis. After 68 days in culture and nine passages, the percentage of putative SSC was 10.1% of a 17-year-old KS patient **(B)**. Several time points were analyzed to evaluate the evolution of this population over time in all subject samples in culture. The graph shows the average of the SSCs population during the culture in KS patients **(B)**.

Successfully propagated testicular cells from KS patients presented putative SSCs at every analyzed time point (**Figure 6**). Quantitative analysis suggested a consistent population in culture, representing between 2-10% of cells in the culture. This indicates that our culture system not only promotes cell propagation, but also provides an excellent environment for maintaining SSCs phenotype, *in vitro*. These results align with prior data using dPCR that identified viable undifferentiated spermatogonia within the culture.

### ID4 positive cells in culture as an SSCs sub-population

**Figure 7:**
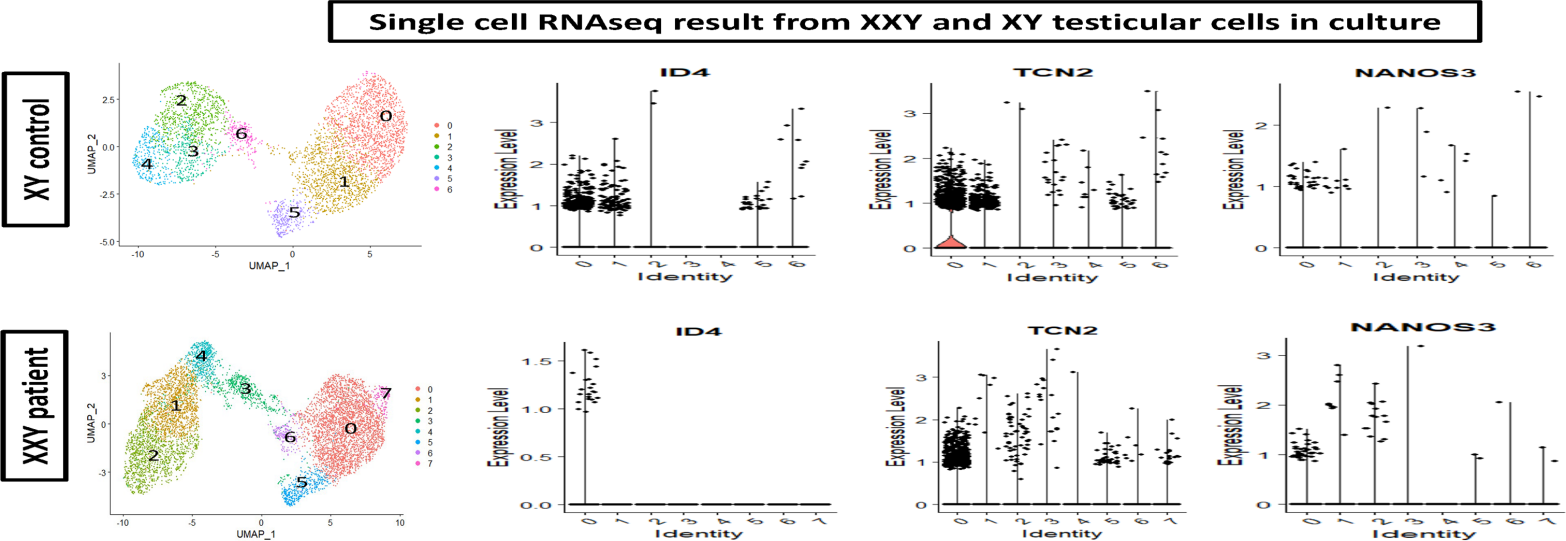
Single cell RNAseq analysis of cultured human XY (upper panel) and XXY (lower panel) testicular cells. Cell partitioning *via* UMAP identified 6 clusters in cultured XY and 7 clusters in cultured XXY testicular cells. Expression patterns of ID4, TCN2 and NANOS 3 (violin plot) supported the presence of SSCs during the culture.

To further characterize the cells in culture, ScRNA Seq analysis was carried out. Cell partitioning *via* UMAP identified 6 clusters in cultured XY and 7 clusters in cultured XXY testicular cells. As shown in **Figure 7**, the ID4, TCN2, and NANOS 3 positive SSCs (46–48) are predominately located within clusters 0 and 1. The data showed that ID4 positive SSCs were more abundant in the XY testicular cell culture than in the XXY culture. The number of TCN2 and NANOS 3 positive SSCs was similar between XY and XXY testicular cell cultures. This data demonstrated that our human testicular cell culture system could provide a favorable environment for SSCs to grow and support survival, *in vitro*.

### The stable genotype of SSCs in culture

NGS technology has been successfully established to assess chromosome copy number and integrity by applying linear regression to the amplification of chromosome-specific sequences for 24-chromosome aneuploidy screening (49). In this study, chromosome copy number analysis was essential to address the concern of genotype instability of cells in culture.

**Figure 8:**
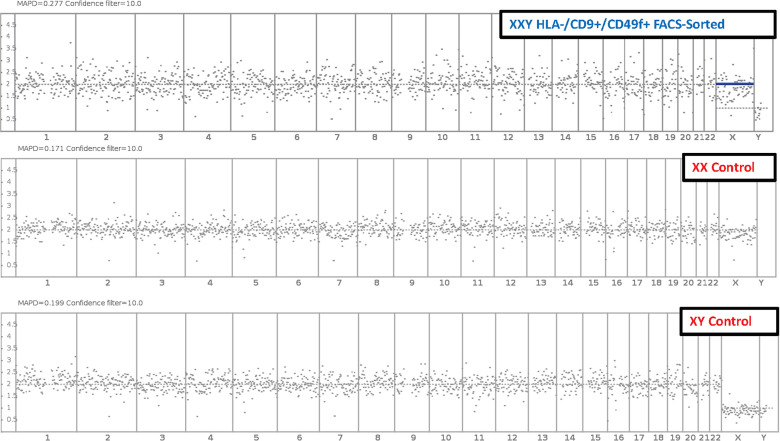
Molecular Karyotyping by next-generation sequencing. After 47 days and 6 passages, Klinefelter cells in culture were FAC Sorted for putative SSC markers HLA-/CD9+/CD49f+. Then cells were analyzed with next generation sequencing and compared to XX and XY controls. Results identified cells in culture as 47 XXY with a 10% confidence filter.

Fluorescence-activated cell sorting (FACS) was used to isolate putative SSCs using the markers HLA^-^ABC. ^-^/CD9 _+_/CD49f ^+^ and compared to peripheral blood XX and XY controls. Results showed no significant difference in the number of somatic chromosomes using up to a 10% confidence filter (**Figure 8**). Sexual chromosome analysis identified an XXY sample compared to XX and XY controls. The sample was finally characterized as non-mosaic 47XXY.

These data suggested that cells in culture remained karyotypically stable. These findings are interpreted as a favorable indication of clinical application potential. The same experiment was conducted on non-sorted cells with equivalent results (data not shown).

### Fluorescent *in situ* hybridization

Fluorescent *in situ* hybridization (FISH) staining for X and Y chromosomes was performed to demonstrate the presence of KS XXY cells in culture. The probes used for this study marked the X chromosome with a red signal (5-ROX) and the Y chromosome with a green signal (5-Fluorescein).

**Figure 9:**
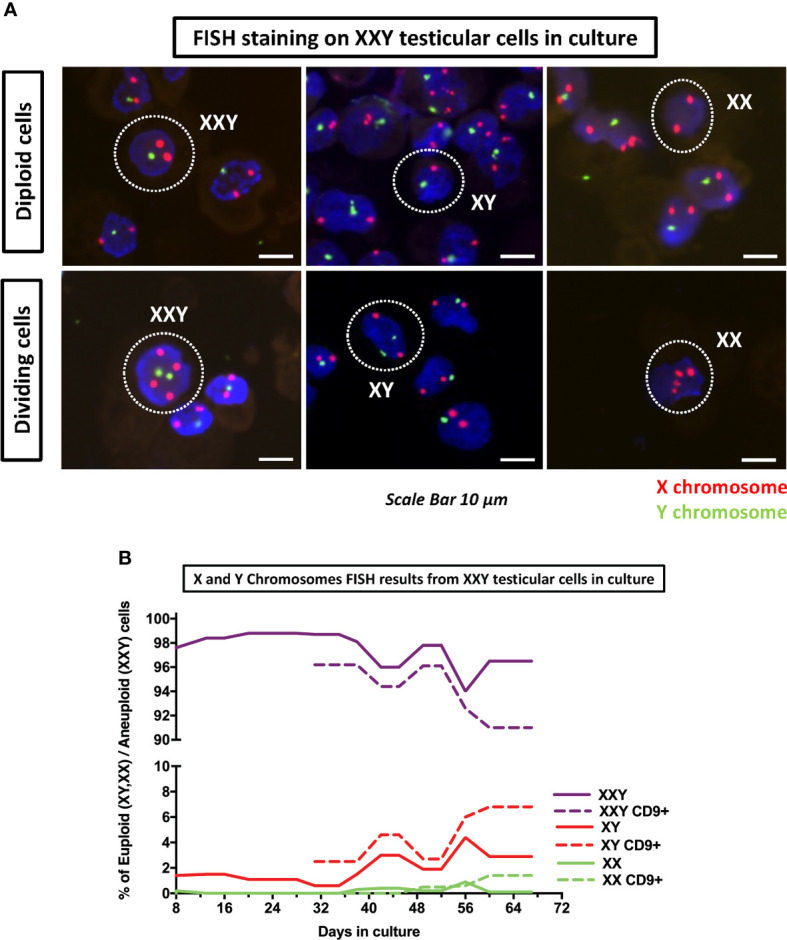
DNA FISH analyses of cultured cells was used to assess the number of copies of X (red probe) and Y (green probe) chromosomes on KS testicular cells in culture. **(A)** Detection of XXY, XY and XX in culture as well as their respective dividing counterparts. **(B)** Quantification of XXY, XY and XX populations in consecutive passages during the culture. The results showed a consistent mosaic population (solid lines). Moreover, when cells in culture were enriched for undifferentiated spermatogonia marker CD9+ using MACS, both XY and XX population increased (dot lines) suggesting that higher number of euploid cells in spermatogonia population.

The initial goal of this analysis was to confirm the aneuploidy of the cells in culture. Cytospun slides were systematically examined, XXY cells were identified. Additionally, XY and XX and their dividing counter partners were also seen (**Figure 9A**).

After these findings were confirmed, we hypothesized that although all of the patients included in this study had been clinically diagnosed as non-mosaic 47XXY Klinefelter, we might have some mosaicism in the testicular cell culture. An experiment was designed to analyze FISH slides in consecutive passages to identify different mosaic populations and describe their evolution in culture over time. Moreover, when the total cell number was sufficient, FISH staining was performed in parallel in CD9+ Magnetic Activated Cell Sorter (MACS) cells and non-sorted controls.

Results showed that even at the earliest time point at four days, there was a small population of both XY (1.4%) and XX (0.2%) (**Figure 9A**). As time passed, both populations thrived and came to represent (4.4%) and (0.9%) respectively. Furthermore, when cells were MACS sorted using CD9 to enrich spermatogonia, XY and XX populations were enriched up to 6.8% and 1.4%, respectively (**Figure 9B**). These findings confirmed that small populations of XY and XX cell mosaicism among XXY cells in culture and suggested XX and XY cells were preferably spermatogonia. This is critical, as euploid spermatogonia may impact the product of spermatogenesis and might, at some level, provide an explanation for the presence of healthy offspring produced from azoospermic KS patients with intratesticular sperm available.

**Figure 10:**
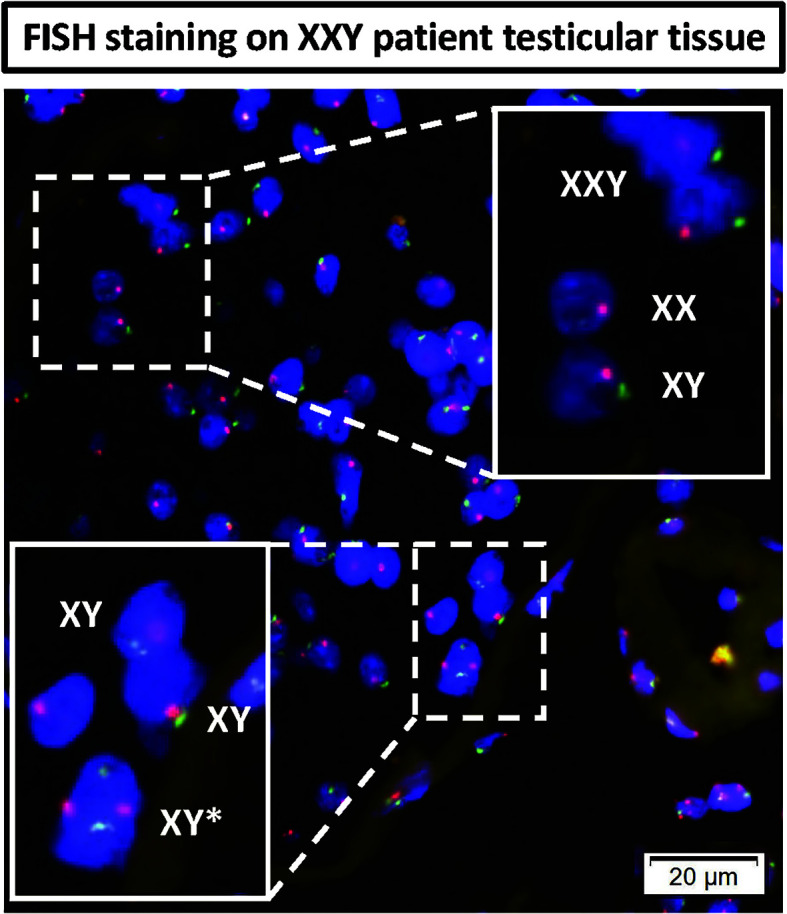
DNA FISH analyses of KS testicular tissue was used to assess any mosaicism in KS testes: X (red probe) and Y (green probe) chromosomes. The top magnified region showed all three XXY, XY and XX cells in non-mosaic KS subject. The bottom magnified region shows an XY cell actively dividing in the basal membrane of seminiferous tubules of non-mosaic KS subject.

A question was raised about the possibility of KS patients presenting some congenital testicular mosaicism that had remained undiagnosed with standard clinical Karyotype techniques. FISH staining was then performed in testicular tissue histology slides from the same patients. The results showed most cells presenting XXY signal. However, few XX and XY cells were found inside the few preserved seminiferous tubules (**Figure 10**). In the basal membrane from one of the tubules, few XY cells were found to be dividing. These findings strongly support some mosaicism in the testes of non-Mosaic 47 XXY KS patients diagnosed by karyotyping.

## Discussion

Although testicular morphology was severely affected by fibrosis in all KS subjects, PGP 9.5 positive spermatogonia cells were still found inside several seminiferous tubules (**Figure 1**). Following isolation and culture of testicular cells from all three subjects, undifferentiated spermatogonia cells were positive for PGP 9.5 ZBTB16 and THY1. These findings suggest that even in patients with negative IHC for spermatogonia, viable spermatogonia are likely present in the cryopreserved samples. Cell isolation and culture under optimal conditions may be able to selectively expand these spermatogonia for fertility treatments. The age of the subjects and other developmental factors may be vital in predicting the success of spermatogonia retrieval and optimizing cell culture.

SSC transplantation is the definitive test for SSCs identification. However, regulatory limitations do not allow for human SSC transplantation. The connection between HLA-/CD9+/CD49f+ enriched cells and SSC transplantation success has long been characterized in autologous animal models and xenotransplantation (37–41). The presence of SSC in our culture system has previous already been tested using xenotransplantation into nude mice (32). We feel that SSC characterization using FACS and RNAseq can reasonably assess the SSC population and may predict SSC transplantation efficacy without the economic costs, ethical challenges, and time associated with low efficient xenotransplantation.

By reaching a 20 million-fold increase in the number of cells in culture, the goal of *in vitro* propagation of spermatogonia cells was achieved. The number of cells isolated per biopsy varied between subjects from 275,000 to 400,000, and the propagation culture system could provide around 5.5 and 8 trillion cells. Conservatively estimating the enriched population of SSCs (HLA-ABC-/CD9+/CD49f+ or ID4 +) as 2% of propagated testicular cells, 110 billion SSCs could be provided after 50 days of culture. Based on previous studies in non-human primates (19, 21, 22), these numbers of cells might potentially enable SSC transplantation. An even lower number of cells might be needed for *in vitro* spermatogenesis (45, 50). Another advantage to this strategy is that only XY SSCs from KS subjects (1% of all propagated SSCs) are likely to go through complete spermatogenesis. Also worth mentioning is that a maximum of 20% of the original testicular biopsy sample was used for this project, as 80% remained un-touched for future clinical applications. When the full samples are used, the starting number of cells in culture will increase, and the duration of culture reduced.

Due to characterization challenges, it has been difficult to quantify the SSC population *in vivo*. However, recent reports by Brinster and Kubota (15) in different mammals estimated this population to be somewhere between 0.01-12.5% of spermatogonia. Using HLA-/CD9+/CD49f+ markers, a population of 2-10% putative SSC was identified in culture. RNAseq data provided confirmation of SSC presence, identifying ID4, TCN2, and NANOS 3 positive clusters of cells. These findings correlate with current literature describing SSC dynamics and their cellular niche, which could indicate an *in vitro* system mimicking *in vivo* physiology (38, 40, 41, 46, 47, 51). Although further research is required on this topic, we expect these data to help optimize the timing between testicular cell culture and SSC transplantation.

Different hypotheses may explain the fluctuation in the number of undifferentiated spermatogonia in culture. It is possible that somatic cells, although able to be maintained in culture long term, do not have the ability to propagate indefinitely. Therefore, somatic testicular cells become less proliferative and efficient in supporting the SSCs population.

FISH and NGS analyses were combined to better characterize the chromosomal stability of cells in culture. Molecular Karyotyping by NGS gave a broad overview of chromosomal integrity across hundreds of thousands of cells using a high number of specific gene loci, very reproducible technology, increased statistical power. The technical limitations of statistical analysis provided a 10% confidence filter, meaning that mosaicisms representing less than 10% of the cells could not be detected.

The limitation of detecting mosaicism in cultured cells was overcome using FISH staining for X and Y chromosomes and manually counting over 5000 cells per subject. Using this method, XY and XX cells were identified within the predominantly XXY population, and the ratio of these karyotypes were characterized during the time in culture. Consistent with NGS data, mosaicism never reached 10%, meaning at least 90% of the cells in culture presented a 47XXY karyotype. Up to 10% might present some mosaicism, including XX and XY karyotypes. Therefore, looking to potential clinical fertility applications, preimplantation genetic screening (PGS) should be recommended for these patients. However, our data support a reasonable level of genetic stability and safety in the cells in culture.

Previous studies have shown sex chromosome mosaicism in cultured iPSCs (52). Similar mosaicism was also described by Hirota et al. (45) using iPSC derived from both XXY mouse and human fibroblasts. Cells in our culture system were not immortalized and achieved long-term culture, up to 100 days. Future studies should compare different cell sources in parallel to verify which options presents the greatest chromosomal stability in culture.

Another interesting finding was that observation of XY and XX cells, even at the earliest timepoint (day four) in our culture system. Similar FISH staining was performed on testis tissue from the same patients before isolation to validate this data. A general review of the slides identifed both XX and XY cells. This fact contradicted previous clinical genetics reports that diagnosed these subjects as non-mosaic Klinefelter 47 XXY. Standard clinical karyotyping usually analyzes no more than 20 cells in peripheral blood smear and cannot rule out organ-specific mosaicism. Although these procedures are beneficial for diagnosing minor defects on the chromosomes constantly present in cells, it lacks the sensitivity to detect small mosaic populations. Further studies are needed to systematically analyze the histology and establish a better quantification method for testicular mosaicism of the KS patient testis, *in vivo*, and its fertility repercussions.

To assess the impact of this finding for future KS fertility treatments, the suitability of KS testis for transplantation should be evaluated. Previous reports by Lue et al. (29) successfully demonstrated SSC transplantation from XY mice into the seminiferous tubules of azoospermic young adult XXY mice. This study suggests that azoospermic KS adult testis, although severely fibrotic, may still retain the basic seminiferous architecture required to perform SSC transplantation. Subsequent studies have shown SSC transplant efficacy in structurally defective seminiferous tubules (53), even without prior germ cell ablation (54) Nevertheless, more studies are needed to confirm that these animal model results are reproducible in humans.

On the other hand, Hirota et al. (45) successfully transplanted primordial iPSC derived germ cell-like cells from XXY mouse fibroblasts into the seminiferous tubules of azoospermic KS mice. Donor XXY SSCs successfully migrated into the basal membrane and restored spermatogenesis. Fertilization and healthy offspring were achieved by ICSI using testicular sperm (45). Our current study suggests that the use of primary human SSCs may provide a better strategy for clinical fertility applications.

## Conclusions

To the best of our knowledge, this is the first report of *in vitro* propagation of human SSCs in long-term culture. This study describes the dynamic chromosomal changes in primary testicular cells from KS subjects in culture. This is a critical step forward in utilizing SSC technology to preserve fertility in KS patients.

## Data availability statement

The datasets presented in this study can be found in online repositories. The names of the repository/repositories and accession number(s) can be found in the article/supplementary material.

## Ethics statement

This study was reviewed and approved by Wake Forest School of Medicine, IRB00021686 and IRB00061265. Written informed consent to participate in this study was provided by the participants’ legal guardian/next of kin.

## Author Contributions

Conceptualization, GG, SK, CW, RS, YL and HS-A. Methodology, GG, YL and HS-A. Software, GG and HS-A. Validation, GG, MP, WK, YL and HS. Formal analysis, GG,ND, WK, YL and HS-A. Investigation, GG,ND, NZ, YL and HS-A. Resources, GG,YL and HS. Data curation, GG,YL and HS-A. Writing—original draft preparation, GG, YL and HS-A. Writing—review and editing, GG, ND, NZ, MP, SK, CW, RS, WK, SH, AA, YL and HS-A. Visualization, GG,YL and HS-A. Supervision, YL and HS-A. Project administration, HS-A. Funding acquisition, AA, YL and HS-A. All authors contributed to the article and approved the submitted version.

## Funding

This work was supported by the Urology Care Foundation Research Scholar Award Program and American Urological Association Southeastern Section. Single cell experiment supported in part by The Lundquist Institute and the UCLA CTSI (ULITR001881-01).

## Acknowledgments

We would like to acknowledge Bethy Jackle, Angela Caviness, Martha ward. Kristine Ali, and Brandi Bickford (Wake Forest diagnostic pathology laboratory) for their technical assistance on FISH staining, automotive immunohistochemistry, and virtual microscopy. We acknowledge the use of tissues procured by the National Disease Research Interchange (NDRI) with support from the NIH grant 5 U42 RR006042”. We also thank Dr Thomas Shupe for editing the manuscript.

## Conflict of interest

The authors declare that the research was conducted in the absence of any commercial or financial relationships that could be construed as a potential conflict of interest.

## Publisher’s note

All claims expressed in this article are solely those of the authors and do not necessarily represent those of their affiliated organizations, or those of the publisher, the editors and the reviewers. Any product that may be evaluated in this article, or claim that may be made by its manufacturer, is not guaranteed or endorsed by the publisher.

## Abbreviations

KS, Klinefelter Syndrome; SSCs, Spermatogonial Stem Cell; SSC, Sodium Chloride-Sodium; dPCR, Digital PCR; MACS, Magnetic Activated Cell Sorting; FISH, Fluorescent *In Situ* Hybridization; PGM, Personal Genome Machine.
